# Clinical observations during adjunctive Cannabis treatment and subsequent oclacitinib dose reduction in dogs with atopic dermatitis

**DOI:** 10.3389/falgy.2026.1852631

**Published:** 2026-10-14

**Authors:** Carollina Mariga, Filipe Costa da Silva, Otávio Henrique de Melo Schiefler, Erik Amazonas, Ana Lucia Souza Silva Mateus, Claudete Schmidt, Saulo Tadeu Lemos Pinto Filho

**Affiliations:** 1 Postgraduate Program in Veterinary Medicine, Federal University of Santa Maria (PPGMV-UFSM), Santa Maria, Rio Grande do Sul, Brazil; 2 Postgraduate Program in Conventional and Integrative Veterinary Medicine (PPGMVCI), Federal University of Santa Catarina (UFSC), Curitibanos, Santa Catarina, Brazil; 3 Department of Statistics, Federal University of Santa Maria (UFSM), Santa Maria, Rio Grande do Sul, Brazil; 4 Department of Small Animal Medicine, Federal University of Santa Maria (UFSM), Santa Maria, Rio Grande do Sul, Brazil

**Keywords:** canine atopic dermatitis, cannabidiol, endocannabinoid system, multimodal therapy, pruritus

## Abstract

Canine atopic dermatitis is a multifactorial chronic skin disease that is difficult to control and requires lifelong and multimodal therapy. Cannabis has regained medical relevance in several fields, particularly in dermatology, as the endocannabinoid system is uniquely expressed in skin tissues and cells. Modulation through cannabinoids aims to restore skin homeostasis by reducing oxidative stress, inflammation, and consequently the clinical signs exhibited by animals with dermatopathies. This study aimed to evaluate Cannabis oil as a therapeutic option for dogs with atopic dermatitis, for both managing recurrent clinical signs and for enabling oclacitinib dose reduction. Sixteen dogs with atopic dermatitis previously treated with oclacitinib without satisfactory improvement were selected. All dogs underwent a fixed-sequence repeated-measures protocol consisting of four standardized evaluation stages: T0 (oclacitinib alone), T30 (placebo + oclacitinib), T60 (Cannabis oil + oclacitinib), and T90 (Cannabis oil + oclacitinib at 50% reduced dose). Dermatological assessments were performed at each stage to monitor clinical progression and response, including CADESI-4, performed by a veterinary dermatologist, and pruritus scoring, reported by the owners; both were blinded to whether the animal was receiving placebo (mineral oil) or Cannabis oil. Of the 16 enrolled dogs, fourteen completed all study stages through T90. No statistically significant difference was observed for CADESI-4. Likewise, no significant difference in pruritus was observed between T30 (placebo) and T60 (Cannabis oil); however, a significant reduction in pruritus was found between T0 x T60 (*p* = 0.0010), and T0 x T90 (*p* = 0.0025). Mild, transient, and self-limiting adverse events, as reported by the owners, were observed during both the placebo and Cannabis oil treatment periods. It is concluded that adjunctive Cannabis oil contributed to improved pruritus control in dogs with atopic dermatitis. In addition, maintenance of pruritus scores was observed following a predefined 50% reduction in oclacitinib dosage, supporting further investigation of Cannabis oil as part of a multimodal therapeutic strategy for canine atopic dermatitis.

## Introduction

Canine atopic dermatitis (cAD) is a multifactorial inflammatory syndrome with a genetic basis, caused by skin barrier dysfunction and an imbalanced immune response involving the dynamic participation of Th1, Th2, Th17, and Th22 inflammatory pathways (1, 2). It is a chronic, lifelong disease that requires treatment focused on improving the skin barrier, modulating the immune response, and preventing secondary infections (3). Due to its lifelong nature, continuous management and multiple therapeutic adjustments are part of the multimodal approach necessary for controlling this syndrome (2). The heterogeneity of clinical responses indicates that monotherapy may be insufficient for some dogs, reinforcing the importance of such multimodal approaches in the management of cAD (4).

Pruritus is one of the most evident clinical signs in atopic dogs and presents a complex pathophysiological mechanism involving concomitant neurological and immunological responses and stimuli (5). Oclacitinib is a Janus kinase (JAK) inhibitor approved for veterinary use, and although it is rapid and effective, refractory cases remain frequent, reinforcing the need for a multimodal therapeutic approach. Moreover, dose adjustments substantially increase treatment costs, limiting its long-term feasibility (1).

The endocannabinoid system (ECS) plays a crucial role in regulating physiological balance by modulating and maintaining organismal homeostasis. The ECS encompasses a complex network with multiple elements that may vary among individuals and tissues (6). Its expression throughout different layers of the skin and in immune system cells suggests that phytocannabinoid supplementation may contribute to the modulation of cutaneous homeostasis, since ECS dysregulation has been associated with inflammatory processes associated with dermatopathies such as cAD (6, 7). Chiocchetti et al. (8) demonstrated that cannabinoids and their receptors expressed in specialized skin cells modulate inflammation and suggest a potential therapeutic approach for dermatopathies. These findings reinforce the importance of the ECS in the pathophysiology of dermatitis, given the increased expression of cannabinoid receptor type 1 (CB1) and cannabinoid receptor type 2 (CB2) receptors in the skin cells of atopic dogs (9) and the attenuation of inflammation observed in animal models through the activation of these receptors (10), suggesting a protective and modulatory role of these components in cutaneous inflammatory processes. Cannabidiol (CBD) has been investigated as an adjuvant therapy for cAD due to its anti-inflammatory and antipruritic properties (1). CBD demonstrates significant immunosuppressive potential by broadly downregulating the Janus kinase/signal transducer and activator of transcription (JAK/STAT) signaling pathway and inhibiting its key activating cytokines, including interleukin (IL) IL-6, IL-2, IL-10, interferon-gamma (IFN-*γ*) and tumor necrosis factor (TNF). Consequently, this targeted cytokine suppression not only dampens JAK/STAT activity but also disrupts interconnected cascades like phosphoinositide 3-kinase/protein kinase B pathway (PI3K-Akt) and mitogen-activated protein kinase pathway (MAPK), thereby attenuating lymphocyte proliferation and overall inflammatory responses (12).

As the ECS may vary among individuals and tissues (6), and Cannabis-based formulations differ between batches due to genetic, environmental, and extraction factors (13), particularities in endocannabinoid tone and entourage effect are frequently observed. Despite the growing interest in this field, there remains a scarcity of controlled clinical trials, and available results are often limited by variability in dosages and formulations (11, 14).

Current evidence regarding cannabinoids in canine atopic dermatitis (cAD) remains contradictory concerning pruritus and lesion scores, and prospective data combining phytocannabinoids with oclacitinib are lacking. This study aimed to bridge this gap by evaluating a standardized adjunctive phytocannabinoid protocol in atopic dogs receiving oclacitinib. We hypothesized that co-administering Cannabis oil would sustain clinical control (pruritus and CADESI-4) during a 50% oclacitinib dose reduction. Although cannabinoids are increasingly explored for canine atopic dermatitis (CAD), reported effects on pruritus score and CADESI-4 scores are variable, and prospective protocols combining cannabinoids with oclacitinib have not been established. Addressing this gap, we investigated an adjunctive full-spectrum Cannabis oil protocol, hypothesizing that it would prevent clinical worsening in pruritus and lesion severity during a structured 50% tapering of oclacitinib.

## Material and methods

### Ethics committee

This study was approved by the Ethics Committee for Animal Use and Experimentation of the Federal University of Santa Maria (CEUA/UFSM) (number 4986140823 - ID 004293) and was conducted in accordance with the ethical principles of the National Council for Animal Experimentation Control (CONCEA).

### Animal selection

Canine subjects with a diagnosis of atopic dermatitis, with prior exclusion of food allergy through an 8–12-week hypoallergenic elimination diet and flea bite dermatitis, were selected according to the criteria of Favrot (15). The inclusion criteria were: AD diagnosis; absence of concurrent diseases; use of oclacitinib as the unique systemic treatment for at least 20 days. The exclusion criteria were: lack of regular flea control; presence of dental calculus and/or moderate-to-severe gingivitis since active periodontal inflammation may alter systemic inflammatory status and represent a potential confounding factor when evaluating the immunomodulatory effects of cannabinoid therapy (16); any other concurrent disease. No maximum or minimum CADESI-4 values were stipulated for selection or exclusion. Additionally, there was no standardization in the use of shampoo for topical treatment or the type of diet used, in order to mimic clinical routine. Although owners were instructed to maintain these interventions unchanged throughout the study period.

The study utilized a prospective, placebo-controlled, repeated measures design, allowing each patient to serve as its own baseline control across sequential treatment phases. Sixteen dogs with a clinical diagnosis of cAD were included in the study (Table 1). In Brazil, the licensed oclacitinib regimen is 0.4–0.6 mg/kg, PO, BID for 14 days, followed by 0.4–0.6 mg/kg, PO, SID as maintenance therapy. All animals had previously received oclacitinib (0.2–1.1 mg/kg, PO, SID) for a minimum period of 20 days (the individualized dose administered to each dog is presented in Table 1). Before study enrollment, the oclacitinib dose was adjusted to maintain a stable and clinically detectable level of pruritus, sufficient to allow owners to perceive treatment-related changes while avoiding excessive pruritus that could compromise animal welfare. All dogs continued to exhibit a clinically detectable degree of pruritus. Owner assessment was used because pruritic behavior was often observed at home, particularly during resting periods and at night, and was not consistently evident during dermatological consultations. No animal achieved complete control of clinical signs within the therapeutic dose range. Following this period, the dogs were admitted in the study and underwent serial dermatological evaluations at four distinct time points: T0 (baseline), T30 (placebo), T60 (Cannabis oil + oclacitinib), and T90 (Cannabis oil + oclacitinib at 50% dose).

**Table 1:** Animals’ demographics.

Animal	Sex	Age	Breed	Age of disease	Previous oclacitinib use	Oclacitinib dose
Animal 1	Male	5 years	Shih-tzu	7 months	First time	0.2 mg/kg
Animal 2	Female	4 years	Shih-tzu	3 years	6 months	0.3 mg/kg
Animal 3	Female	7 years	Shih-tzu	1 year	6 months	0.6 mg/kg
Animal 4	Female	7 years	Shih-tzu	1 year	4 years	0.6 mg/kg
Animal 5	Male	1 year	Maltese	6 months	First time	0.2 mg/kg
Animal 6	Male	9 years	Shih-tzu	3 years	First time	0.2 mg/kg
Animal 7	Female	4 years	Shih-tzu	1 year	3 years	0.6 mg/kg
Animal 8	Female	5 years	Pekingese	10 months	6 months	0.2 mg/kg
Animal 9	Male	5 years	Chihuahua	2 years	First time	1.1 mg/kg
Animal 10	Female	3 years	Shih-tzu	2 years	9 months	0.3 mg/kg
Animal 11	Female	8 years	Shih-tzu	3 months	2 years	0.6 mg/kg
Animal 12	Female	1 year	Shih-tzu	3 months	First time	0.9 mg/kg
Animal 13	Female	7 years	Shih-tzu	1 year	First time	0.4 mg/kg
Animal 14	Female	7 years	Shih-tzu	3 months	1 year	0.5 mg/kg
Animal 15	Male	6 years	Shih-tzu	2 months	6 months	0.5 mg/kg
Animal 16	Male	12 years	Shih-tzu	Unknown^a^	6 months	0.5 mg/kg

a Adopted as an adult, with pre-existing skin problems; “First time” refers to dogs receiving oclacitinib for the first time in their medical history, as opposed to dogs with prior chronic/intermittent use. All enrolled subjects received daily oclacitinib for a minimum of 20 days prior to baseline evaluation (T0).

The experimental protocol was standardized for all dogs and followed the sequence described below (Figure 1):

**Figure 1:**
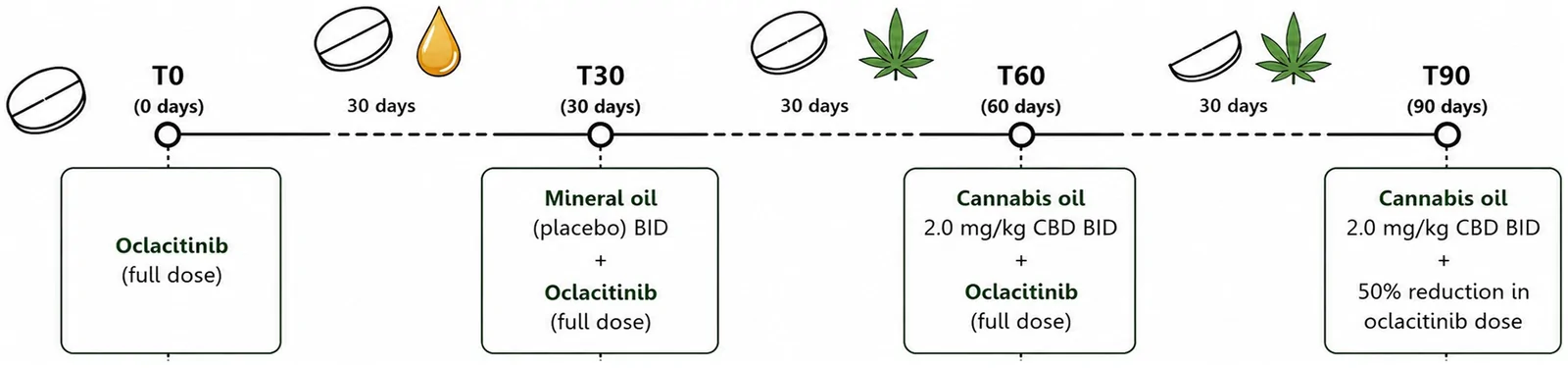
Study design and treatment timeline. Sixteen dogs with canine atopic dermatitis previously treated with oclacitinib without satisfactory clinical control were enrolled. All animals followed the same fixed treatment sequence: at baseline (T0), dogs received oclacitinib alone; After 30 days (T30), mineral oil (placebo) was administered twice daily (BID) in association with the full dose of oclacitinib; At T60, placebo was replaced with Cannabis oil (2 mg/kg of CBD, BID) while maintaining the full oclacitinib dose; At T90, Cannabis oil administration was maintained and the oclacitinib dose was reduced by 50%. Dermatological assessments, including CADESI-4 and pruritus score, were performed at all evaluation time points.

T0 (Baseline – Oclacitinib only): initial stage of the study, during which dogs were receiving oclacitinib [0.2–1.1 mg/kg, once a day (SID)] alone for at least 20 days before the baseline assessment, at individualized doses within an off-label range. A baseline dermatological evaluation was performed at this time point.

T30 (Placebo + Oclacitinib): during the following 30 days, mineral oil was administered orally (PO), twice a day (BID), in combination with oclacitinib at the full dose. A new dermatological evaluation was performed at the end of this period.

T60 (Cannabis oil + Oclacitinib): dogs received Cannabis oil at a dose of 2.0 mg/kg of CBD, PO, BID, in association with oclacitinib at the full dose SID, for 30 days. A subsequent dermatological evaluation was then conducted.

T90 (Cannabis oil + Reduced Oclacitinib): administration of Cannabis oil (2.0 mg/kg of CBD, PO, BID) was maintained, while the oclacitinib dose was reduced by 50% of the initial dose. After 30 days, the final dermatological evaluation was performed.

Thus, all animals underwent the same treatment stages in the following fixed chronological order: T0 (oclacitinib alone) → T30 (placebo) → T60 (Cannabis oil + oclacitinib) → T90 (Cannabis oil + oclacitinib at 50%). The oclacitinib dose was adjusted to standardize a minimal degree of pruritus prior to the study. Although ethically feasible parallel control designs, such as full-dose oclacitinib plus placebo, could have been considered, the present study was specifically designed as a repeated-measures dose-reduction protocol, with each dog serving as its own control. Thus, each animal served as its own control, which is also methodologically appropriate due to the marked interindividual heterogeneity in the tone and expression of components of the ECS (6). This design reduces individual biological variability and allows for a more precise evaluation of therapeutic effects over time within the same dog.

### Dermatological evaluation

The animal was assessed by a dermatologist using the Canine Atopic Dermatitis Extent and Severity Index CADESI-4 score (17), adapted to include evaluation of the ventral cervical region based on its clinical relevance in dogs with cAD, and according to the owner, the itching degree (18), which ranges from zero to 5, with higher scores indicating greater pruritus severity. The Marsella scale was selected because it provides a simple owner-reported assessment of pruritus severity based on observable behaviors, facilitating consistent evaluation throughout the study period. The same owner completed all pruritus assessments for each dog at every evaluation time point. Both the dermatologist and the owner were blinded to the identity of the experimental oil administered during each phase of the study (placebo mineral oil or Cannabis oil). The dermatologist was not informed of the study design or treatment sequence.

Mineral oil was selected as placebo because it is considered pharmacologically inert in relation to skin inflammation and pruritus. Artificial yellow food coloring was added to mimic the appearance of the Cannabis oil.

In addition to dermatological assessments, owners were questioned at each study visit regarding the occurrence of potential adverse effects during the preceding treatment period. Following completion of the study, owners were also contacted to document clinical evolution after discontinuation of Cannabis oil, including recurrence of pruritus, maintenance of disease control, and continuation of cannabinoid therapy.

### Acquisition of Cannabis oil

A full-spectrum Cannabis oil rich in cannabidiol (CBD) (500 mg/30 mL) was used, donated by *Hippie Hounds® Treats*, under the Brazilian simplified special authorization for educational and Research institutions (AEP No. 20/2022 and 004/2025) and authorization for the importation of controlled substances and medications (AI/151/2023), both approved by the National Health Surveillance Agency (ANVISA/MS). The product underwent laboratory analysis performed by *F.A.S.T. Laboratories/Research* (Sample ID: S128929) and is available as Supplementary Material.

### Statistical analysis

All statistical analyses were performed using R software (version 4.3.1), with a two-sided significance level set at 5%. Specifically, cumulative link mixed models and model adequacy were evaluated using the ordinal package (19), simulation-based residual diagnostics were conducted using the DHARMa package (20), and pairwise comparisons were carried out using the emmeans package (21).

### CADESI

Changes in CADESI-4 scores over time were evaluated using generalized linear mixed models (GLMMs). Because CADESI-4 is a discrete, non-negative composite score with a potentially asymmetric distribution and possible overdispersion, different response-distribution specifications were evaluated, including Poisson, negative binomial type 1 (NB1), negative binomial type 2 (NB2), and zero-inflated negative binomial models (ZINB1 and ZINB2). All models included treatment stage (T0, T30, T60, and T90) as a fixed effect and animal identification as a random intercept to account for the correlation among repeated measurements within the same animal.

Model selection was based jointly on the Akaike information criterion (AIC), Bayesian information criterion (BIC), and simulation-based residual diagnostics, including tests for dispersion, zero inflation, and detection of outlying observations.

The primary planned contrast was T30 versus T60, while the second planned contrast was T60 versus T90. These contrasts were defined *a priori* according to the main clinical questions addressed by the study. Comparisons involving the baseline period (T0) and subsequent treatment stages were considered secondary, as they reflect differences in both treatment exposure and calendar time. Pairwise comparisons were reported as ratios of expected mean scores, together with their corresponding 95% confidence intervals and Holm-adjusted *p*-values.

### Pruritus degree

Longitudinal pruritus scores were analyzed using a cumulative link mixed model (CLMM), fitted with the clmm function from the ordinal package, accounting for the ordinal categorical nature of the scale and the repeated-measures study design. A logit link function was used.

The model included treatment stage (T0, T30, T60, and T90) as a categorical fixed effect and animal identification as a random intercept. Model adequacy was assessed with respect to the proportional-odds assumption, the distribution of the random effects, and the behavior of predicted values and residuals. The distribution of the scores was also examined *a priori*, considering the frequency of the observed categories and the presence of categories with low frequencies.

The proportional-odds assumption was evaluated using a likelihood ratio test (LRT), comparing the proportional-odds model with a more flexible specification. The absence of evidence for a violation of this assumption was considered consistent with the use of the proportional-odds model.

The primary planned contrasts were T30 versus T60 and T60 versus T90, defined *a priori* according to the clinical questions addressed by the study. Comparisons involving baseline (T0) and subsequent treatment stages were considered secondary. Pairwise contrasts among time points (T0–T30, T0–T60, T0–T90, T30–T60, T30–T90, and T60–T90) were expressed as odds ratios (ORs), with corresponding 95% confidence intervals (95% CIs), applying Tukey's adjustment for multiple comparisons. ORs < 1 were interpreted as lower cumulative odds of being in higher pruritus categories compared with the reference time point.

## Results

The generalized linear mixed model with a type 1 negative binomial distribution showed no significant effect of time on CADESI-4 scores (Likelihood Ratio Test: *χ*^2^ = 4.66, df = 3, *p* = 0.198). The estimated marginal mean scores were 4.65 at T0, 5.13 at T30, 2.74 at T60, and 3.59 at T90. The primary planned contrasts T30 versus T60 comparison, showed no statistically significant difference (ratio = 1.872; 95% CI: 0.931–3.760; *p* = 0.0882). Similarly, the T60 versus T90 comparison showed no statistically significant difference (ratio = 0.765; 95% CI: 0.347–1.680; *p* = 0.4467). Secondary comparisons between baseline (T0) and subsequent treatment stages also revealed no statistically significant differences, including T0 versus T30 (ratio = 0.906; 95% CI: 0.472–1.74; *p* = 0.8349), T0 versus T60 (ratio = 1.696; 95% CI: 0.782–3.68; *p* = 0.3070), and T0 versus T90 (ratio = 1.297; 95% CI: 0.602–2.80; *p* = 0.8349). After Holm adjustment for multiple comparisons, no statistically significant differences were detected in any of the evaluated pairwise comparisons (all *p* > 0.05). Figure 2 presents box plots for each treatment period, along with lines illustrating the individual trajectories of each treated dog.

**Figure 2:**
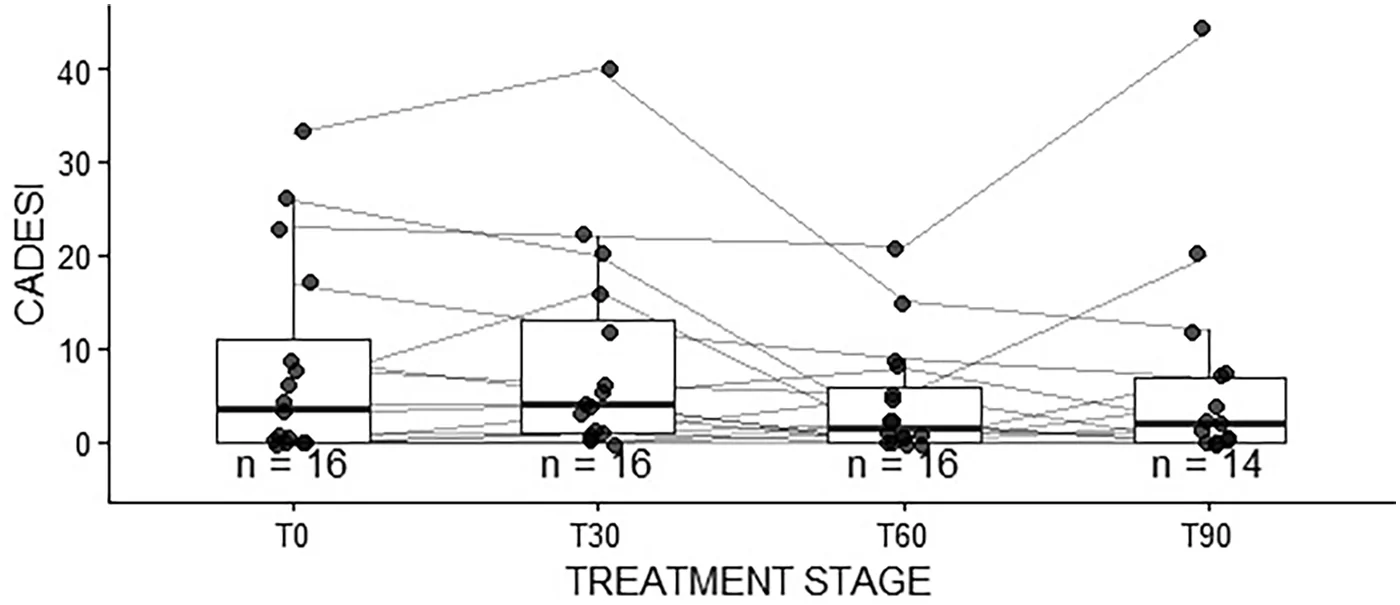
Box plots representing dermatological assessment using CADESI-4 across different treatment stages (T0, T30, T60, and T90). Boxes represent the interquartile range, horizontal lines indicate the median, and whiskers extend to the most extreme observations within 1.5 × IQR. Individual points represent CADESI-4 scores for each dog at the corresponding evaluation time point. Lines connect repeated measurements from the same dog across treatment stages, illustrating individual trajectories over time. No statistically significant differences were detected among the evaluated time points (*p* > 0.05).

Pruritus scores were additionally analyzed using a cumulative link mixed model (CLMM), accounting for the ordinal nature of the pruritus scale and the repeated measurements within animals. The model showed a significant effect of time on pruritus scores (Likelihood Ratio Test: *χ*^2^ = 24.48, df = 3, *p* < 0.001). The estimated marginal mean pruritus scores were 3.44 at T0, 3.00 at T30, 2.31 at T60, and 2.42 at T90. The distribution of observed pruritus scores and individual trajectories across treatment stages are presented in Figure 3. The primary planned contrasts T30 versus T60 comparison showed no statistically significant difference (estimate = 1.3499; OR = 5.50; 95% CI: 0.85–35.67; *p* = 0.0889). Similarly, the T60 versus T90 comparison showed no statistically significant difference (estimate = −0.3523; OR = 0.70; 95% CI: 0.11–4.41; *p* = 0.9607), after Tukey adjustment for multiple comparisons. Secondary, compared with T0, no statistically significant difference was observed at T30 (estimate = 1.3499; OR = 3.86; 95% CI: 0.58–25.70; *p* = 0.2597). In contrast, statistically significant reductions in pruritus were observed at T60 (estimate = 3.0541; OR = 21.20; 95% CI: 2.53–177.48; *p* = 0.0013) and T90 (estimate = 2.7018; OR = 14.91; 95% CI: 1.76–126.58; *p* = 0.0065) compared with baseline. These results indicate lower cumulative odds of being in higher pruritus categories at T60 and T90 compared with baseline. The additional secondary comparison between T30 and T90 also showed no statistically significant difference (estimate = 1.3519; OR = 3.86; 95% CI: 0.57–26.25; *p* = 0.2672), after Tukey adjustment for multiple comparisons.

**Figure 3:**
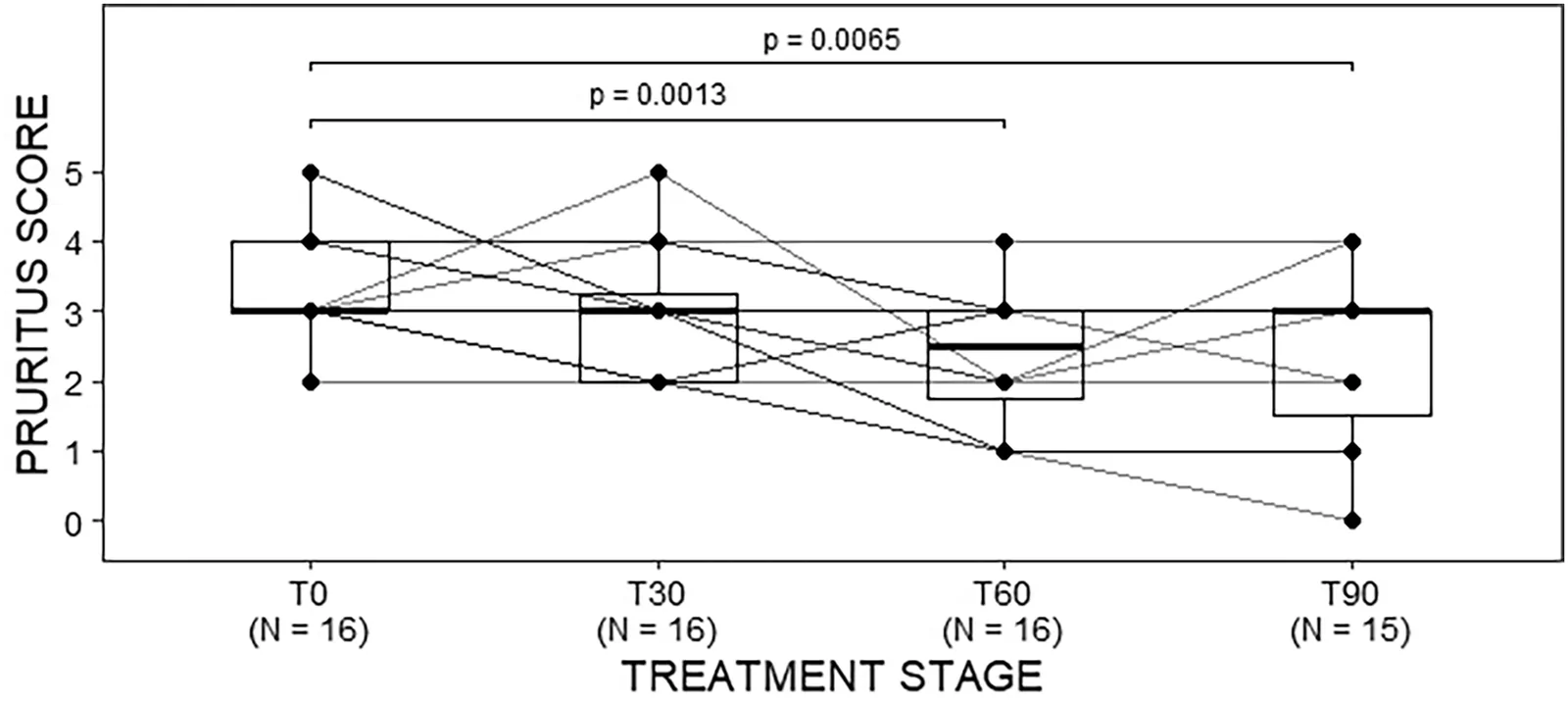
Box plots representing the comparison of pruritus scores across different treatment stages [T0 vs. T60; T0 vs. T90]. Box plot elements are described in Figure 2. Individual points represent pruritus scores for each dog at the corresponding evaluation time point. Lines connect repeated measurements from the same dog across treatment stages, illustrating individual trajectories over time. Statistically significant differences were observed between T0 and T60 and between T0 and T90 (*p* < 0.05).

After the final scheduled evaluation (T90), the Cannabis oil was interrupted while maintaining 50% of the oclacitinib dose; Dogs were re-evaluated 7 days later, during this period, nine animals resumed scratching within one week: four on the second day, two on the third day, one on the fourth day, and two on the seventh day. The remaining five animals did not show pruritus after the first seven days without Cannabis oil, remaining stable on 50% of the oclacitinib dose. Two animals were excluded from the study before completing the final month of treatment (T90) due to deep secondary skin infections requiring systemic therapy, diagnosed clinically and by skin cytology. Nine owners (56.25%) chose to continue Cannabis treatment after participation in the study.

Regarding adverse effects, during the first month (*N* = 16) of treatment (placebo), 10 animals (62.5%) experienced at least one adverse clinical sign, including four animals (25%) presented with drowsiness, soft stools, and vomiting. In the second month (*N* = 16) of treatment (introduction of Cannabis oil), 11 animals (68.75%) experienced at least one adverse clinical sign, including five animals (31.25%) exhibited drowsiness, four (25%) vomiting, two (12.5%) softening of stools, and one (6.25%) agitation. In the final month (*N* = 14) of treatment (maintenance of Cannabis oil combined with 50% reduction of the conventional medication dose), five animals (35.71%) experienced at least one adverse clinical sign, including three animals (21.43%) showed drowsiness, one (7.14%) had soft stools, and one presented vomiting. Dogs could present with more than one clinical sign per treatment period. All reported adverse events were mild, transient, and self-limiting.

## Discussion

The present study evaluated the effects of adjunctive Cannabis oil administration in dogs with atopic dermatitis receiving oclacitinib, with particular emphasis on clinical pruritus and dermatological lesions during a progressive reduction in the conventional treatment. Pruritus scores showed a significant reduction compared with baseline, with significant differences between T0 and T60 and between T0 and T90. However, the direct comparison between the placebo phase (T30) and Cannabis treatment (T60) did not reach statistical significance, and no significant worsening of pruritus was observed between T60 and T90 despite the 50% reduction in the oclacitinib dose. No significant changes were observed in CADESI-4 scores across the evaluated time points. Although not statistically significant, clinical improvement in skin lesions was observed in some animals (Figure 4).

**Figure 4:**
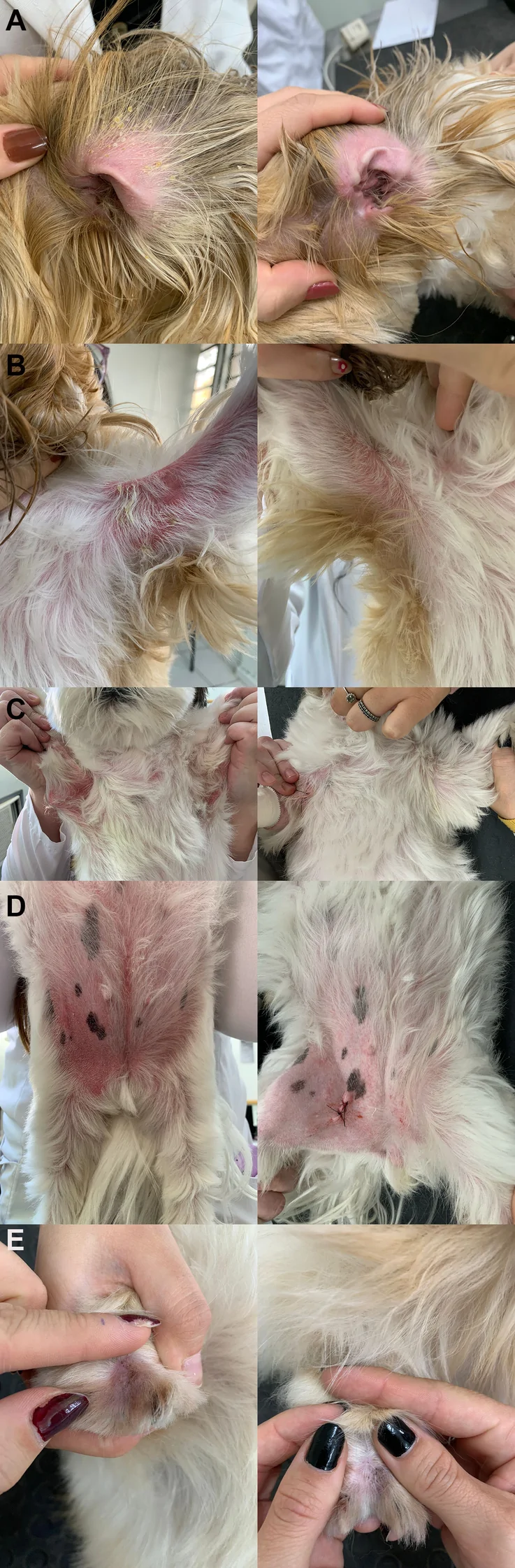
Representative clinical photographs illustrating changes in skin lesions between T30 and T60 in five dogs with canine atopic dermatitis. Each letter represents an individual dog, with T30 shown on the left and T60 on the right: **(A)** auricular region; **(B)** axillary region; **(C)** ventral thoracic region; **(D)** inguinal region; and **(E)** interdigital region. At T30, dogs received placebo (mineral oil) in combination with the full oclacitinib dose, whereas at T60, dogs received full-spectrum, CBD-rich *Cannabis* oil in combination with the full oclacitinib dose. Compared with T30, the photographs at T60 show visible changes in erythema, scaling, crusting, and other inflammatory skin lesions in these representative animals. These qualitative clinical observations complement the individual CADESI-4 trajectories presented in Table 2; however, changes in CADESI-4 were not statistically significant at the group level.

**Table 2:** Pre-treatment (T0), placebo (T30), post-treatment with 2.0 mg/kg of full-spectrum high cannabidiol oil (T60), and maintenance of 2.0 mg/kg of full-spectrum high cannabidiol oil with reduction 50% of oclacitinib dose (T90) CADESI-4 and itching degree evaluation in dogs with atopic dermatitis.

Animal	CADESI-4				Itching degree (0–5)			
	T0	T30	T60	T90	T0	T30	T60	T90
Animal 1	9^a^	5^a^	8	2^a^	3	3	1	1
Animal 2	17^a^	12	9	7	3	2	3	2
Animal 3	33	40	15	12	5	3	3	3
Animal 4	1^a^	1	5	20^b^	3	3	2	4
Animal 5	0	0	2	0	3	3	2	2
Animal 6	0	1	2	0	4	3	1	0
Animal 7	8^a^	6	5^a^	0^a^	3	2	3	3
Animal 8	0	0	1	1^a^	3	2	1	1
Animal 9	0	0	0	4^a^	2	2	2	3
Animal 10	6	16^a^	0^a^	0^a^	3	4	3	3
Animal 11	26	20	1^a^	.	4	4	3	.
Animal 12	0	1	0	2	3	2	1	1
Animal 13	4	4	0	0	4	4	4	4
Animal 14	23	22	21	44	4	3	3	3
Animal 15	0	3	0	.	3	5	2	.
Animal 16	3	4	0	7	5	3	3	3

a Presence of lesions not evaluated by CADESI-4 observed by the dermatologist: epidermal collarette, pustules, hyperpigmentation, scaling, xerosis, blepharitis, heterotrichia, and/or otitis externa (the latter observed only at T90).

b Absence of xerosis; T90 data are unavailable for Animals 11 and 15 because both were withdrawn from the study before the final evaluation due to secondary skin infections requiring systemic therapy.

The biological plausibility of an antipruritic effect of Cannabis may be related to the role of the ECS in cutaneous homeostasis. The ECS has gained recognition as an important and multifaceted homeostatic regulator, deeply involved in maintaining skin homeostasis, in the formation and regeneration of the skin barrier, and its dysregulation has been associated with the development of dermatopathies such as atopic dermatitis (7). Genetically modified mice lacking CB1 receptors in primary sensory neurons exhibited exacerbated inflammation and pruritus (22). This deletion promoted substance P production, which affected extracellular signal-regulated kinase (ERK) activation in keratinocytes, inducing mast cell accumulation in the dermis. Mice with CB2 receptor deletion displayed exacerbated psoriasiform dermatitis and pruritus compared to normal mice (23). The significant reduction in clinical pruritus observed in the present study may be related to mechanisms described in the literature, particularly those involving modulation of cannabinoid receptors and the cutaneous neuroimmune response (22–24). These mechanisms provide a biologically plausible basis for the potential antipruritic effects of Cannabis. However, as these pathways were not directly evaluated in the present study, they should be interpreted as potential mechanisms underlying the observed clinical effect rather than as mechanisms demonstrated by our findings.

The present study demonstrated no significant improvement in dermatological evaluation across the compared treatments. These findings are consistent with those reported by Loewinger et al. (25), who conducted a prospective, randomized, double-blinded, placebo-controlled study involving 32 dogs with atopic dermatitis treated with 2.0 mg/kg of a CBD/CBDA (Cannabidiolic acid) mixture or placebo for four weeks. The authors observed a significant reduction in pruritus (*p* = 0.01), although the difference compared to the placebo group did not reach statistical significance (*p* = 0.054), and no improvement was detected in skin lesions assessed by CADESI-4. Similarly, in the present study, although a significant reduction in pruritus was observed relative to baseline (T0) the direct comparison between the placebo phase (T30) and the Cannabis treatment phase (T60) did not reach statistical significance. Thus, both studies showed reductions in pruritus over the course of treatment, but neither demonstrated a statistically significant difference in pruritus between the cannabinoid and placebo conditions. In contrast, the retrospective study by Mogi et al. (26) reported improvement in CADESI-4 and pruritus scores in eight dogs with atopic dermatitis treated twice daily for at least eight weeks with a CBD-rich oil. However, these eight dogs were not compared to a control group, and the doses varied from 0.14–1.43 mg/kg/day, increased gradually according to each patient´s clinical response. It is worth noting that in our present study the cannabinoid dosage was not increased to compensate for the 50% reduction in oclacitinib, and the formulation used contained predominantly CBD (CBD 73.7%, THC 2.11%, CBC 2.05%, CBG 1.85, and other minor phytocannabinoids), expressed as relative proportions of the total cannabinoid mass analyzed. Cannabis products with a higher proportion of other cannabinoids such as *Δ*9-tetrahydrocannabinol (THC) could potentially elicit greater clinical effects, given the expression of CB1 receptors in keratinocytes and cutaneous nerve endings, which play a role in neuroimmune modulation and inflammation control (5, 27). The therapeutic response could potentially have been further enhanced had the administered formulation also contained higher proportions of the minor phytocannabinoids cannabichromene (CBC) and cannabigerol (CBG). Hong et al. (28) demonstrated that CBC downregulates master inflammatory signaling by simultaneously inhibiting the NF-kB and MAPK pathways, significantly suppressing downstream effector cytokines (TNF-α, IL-1β, and IL-6) and inducible nitric oxide synthase (iNOS). Li et al. (29) established that CBG provides multifaceted immunomodulation via CB2 receptor engagement, PPAR-γ and Nrf2 activation, and direct inhibition of inflammatory enzymes (COX-1, COX-2, and iNOS), thereby attenuating pro-inflammatory mediators (TNF-α, IL-1β, and IL-6, and IFN-γ) as well as reactive nitrogen and oxygen species. The complex immunopathology of cAD includes persistent leukocyte infiltration, oxidative damage, and neurogenic itch that perpetuate chronic epidermal dysfunction, thus tailored phytocannabinoid profiles with elevated CBC and CBG represent a promising avenue to optimize clinical remission, accelerate barrier repair, and further reduce chronic erythema and lichenification in atopic dogs.

In the present study, significant reductions in pruritus scores were observed between T0 and T60 and between T0 and T90. Similar findings were reported by Loewinger et al. (25), who observed a significant reduction in pruritus following treatment with a CBD/CBDA formulation, despite the absence of improvement in CADESI-4 scores. In contrast, Mariga et al. (30) did not detect significant differences in either pruritus scores, CADESI-4 or histopathological mast cell count in skin biopsies compared in dogs with atopic dermatitis treated with Cannabis oil compared with placebo.

To the best of our knowledge, this is the first study to report the association of Cannabis oil and oclacitinib in dogs with cAD and to provide a preliminary observation of a potential drug-sparing effect associated with oclacitinib dose reduction. In reference to the results obtained in the present study, a 50% reduction in the oclacitinib dose along with cannabinoid supplementation was not associated with a significant worsening of pruritus or skin lesions compared with the preceding treatment phase (cannabinoids + oclacitinib at full dose). Case reports such as those by Mariga et al. (31) and Mogi et al. (32) indicate that therapy with Cannabis oil may allow the gradual reduction of conventional medications, such as cyclosporine and corticosteroids, while maintaining control of pruritus and promoting improvement of skin lesions. These findings reinforce the potential of Cannabis as an adjuvant therapy, even considering the methodological limitations typical of case reports, and highlight the need for further controlled clinical studies to consolidate these observations.

Comparing the demographic profile described by Mariga et al. (30), the mean disease duration reported in the treated animals was 5.6 years, whereas in the present study, the mean age was only three years. This study, similar to Mariga et al. (30), did not establish minimum or maximum values for skin lesions according to CADESI-4; however, the lesion scores observed here were considerably lower [T0: mean = 8.1 (0 ± 33); T30: mean =8.4 (0 ± 40); T60: mean =4.3 (0 ± 21); T90: mean =7.0 (0 ± 44)] compared to those reported in the 2023a study [Pre-treatment: mean =36.7 (7 ± 82); post-treatment: mean =31.9 (7 ± 114)]. This difference reflects our decision to include dogs across a wider range of disease severity, rather than focusing predominantly on animals with more advanced dermatological lesions as in our previous study. Consequently, several dogs presented with low or null baseline CADESI-4 scores, which may have reduced the sensitivity of this instrument to detect treatment-related improvements. It is noteworthy that the animals in the present study received cannabinoid therapy at an early stage of the disease. Although the present findings do not support a recommendation of Cannabis as a first-line treatment, they raise the hypothesis that earlier introduction of cannabinoid therapy may offer clinical benefits before chronic inflammatory changes become fully established. However, this hypothesis remains strictly exploratory given the methodological constraints of the present study, as the timing of intervention was not a predefined independent variable and no comparative assessment between early and advanced disease stages was conducted. Nonetheless, supported by our prior prospective investigations in canine atopic dermatitis and cumulative observations in veterinary cannabinoid therapy, this concept represents a biologically plausible framework that warrants reporting to inform future clinical trials aimed at early-stage disease modulation. This interpretation is further supported by the observations of Mogi et al. (26), who highlighted the potential importance of early phytocannabinoid supplementation, as its use as a sole therapy in refractory or severe cases does not result in significant clinical improvement. The same authors also emphasized the benefits of combining cannabinoids with conventional drugs, as this approach may allow dose reduction and consequently enhance overall treatment efficacy. Although CADESI-4 assesses both acute and chronic lesions, chronic lesions are more extensive and persistent, and yield higher scores, thus tending to dominate the overall score in dogs with long-standing atopic dermatitis.

The two animals excluded from the present study before completing the third month of treatment were due to the presence of secondary infection requiring therapy. Importantly, these events occurred during the final stage of the study protocol, after completion of the previous evaluation periods. These animals (n°. 11 and n°. 15) had demonstrated favorable clinical evolution throughout the study period prior to withdrawal, including low CADESI-4 scores and improvement in pruritus assessments.

In contrast, some animals remained in the study despite exhibiting higher lesion scores. Animals n°. 4 and n° 14 had been undergoing treatment with oclacitinib for 4 and 1 year, respectively, with animal number 4 being the one with the longest treatment duration in this study. It is noteworthy that both animals were receiving oclacitinib at the recommended therapeutic dose. While oclacitinib effectively targets IL-31 and specific Th2-driven signaling, it does not comprehensively suppress all inflammatory pathways, leaving chronic inflammation and underlying epidermal barrier dysfunction partially unaddressed (1). Given the multifactorial and progressive nature of cAD, the ​​gradual loss of clinical control over time may be attributed to continuous disease progression, environmental triggers, and potentially insufficient control following the 50% reduction in oclacitinib during the final treatment phase, rather than being attributed to a single factor (4). The marked increase in CADESI-4 observed in these two animals at T90 highlights the interindividual variability in clinical response and should be considered when interpreting the group-level findings.

The entourage effect is a synergistic phenomenon resulting from the interaction between phytocannabinoids and other molecules present in Cannabis, such as terpenes and polyphenols. Although research often focuses on CBD and THC, *in vitro* studies have demonstrated that extracts containing multiple phytocannabinoids can act integratively on inflammatory pathways such as MAPK, NF-κB, NLRP3 (inflammasome), Janus kinase 1/signal transducer and activator of transcription 6 (JAK1/STAT6) signaling pathway, thereby modulating the immune response of skin cells (33, 34). The full-spectrum Cannabis oil used in this study contains mainly CBD (73.7%) but also includes *Δ*9-tetrahydrocannabinol (*Δ*9-THC, 2.11%), cannabidivarin (CBDV, 0.69%), cannabichromene (CBC, 2.05%), cannabigerol (CBG, 1.85%), cannabigerolic acid (CBGA, 0.116%) and cannabinol (CBN, 0.297%), as well as terpenes such as guaiol, caryophyllene, nerolidol, humulene, and bisabolol – composing a chemical profile capable of promoting multitarget effects. Our *in vivo* findings are consistent with the observations reported by Han et al. (33), particularly regarding the proposed roles of CBD and CBGA in modulating skin inflammation, and those of Di Meo (35) concerning CBN, which prevents keratinocyte inflammation via transient receptor potential vanilloid 1 (TRPV1) activation. However, one study compared the *in vitro* effects of isolated or combined exposure to different concentrations of CBD, CBDA, and β-caryophyllene in lymphocytes from healthy and atopic dogs and found no anti-inflammatory effect (36). Considering that variability in CBD concentrations and formulations may influence the efficacy observed across different clinical studies in atopic dogs, as described by Bizarro et al. (11), detailed characterization of the oil used is essential to better understand the entourage effect exhibited by this remarkably complex plant.

In the present study, the main adverse effects observed were drowsiness, vomiting, and soft stools. All episodes were mild, self-limiting, and occurred only once during the study period. Drowsiness was the most frequent adverse effect, which is consistent with previous reports evaluating Cannabis-derived products in dogs, where sedation was commonly observed and gastrointestinal disturbances were generally described as mild and transient (37, 38). Interestingly, 20% of the animals also presented drowsiness, vomiting, or soft stools during the 30-day placebo period. Although studies evaluating placebo effects in dogs are limited, this finding may be partially explained by the caregiver placebo effect, in which owners may attribute expected adverse effects to treatment after being informed about the study procedures and potential outcomes, even when the animal is not receiving cannabinoid therapy (39). However, spontaneous occurrence of gastrointestinal signs, as well as possible effects related to the mineral oil or food coloring used in the placebo formulation, should also be considered as alternative explanations. Interestingly, CBD and low to moderate doses of THC are generally associated with antiemetic and anti-nausea effects in the literature (40). Similarly, no relevant behavioral alterations were identified throughout the treatment period. Similar findings have been reported by Morris et al. (41), who observed no significant changes in daily activity following CBD administration in healthy dogs, and by Mariga et al. (42), who found no behavioral alterations after 60 days of treatment with a full-spectrum CBD-rich Cannabis oil.

Several studies indicate that individual variability in the ECS and the heterogeneity of Cannabis formulations represent major limitations in research on cannabinoid therapy (6, 11, 13, 43). This variability may arise from interindividual differences in the expression and distribution of cannabinoid receptors, the activity of enzymes involved in endocannabinoid synthesis and degradation, and baseline endocannabinoid levels, which can influence pharmacological responses to cannabinoid-based treatments (6). To address these issues, in the present study, each animal served as its own control, allowing for a more precise evaluation of individual clinical responses. Furthermore, the formulation used underwent rigorous laboratory analysis (Supplementary Material), confirming its Cannabinoid and terpene composition and ensuring the absence of contaminants such as heavy metals, pathogenic microorganisms, mycotoxins, pesticides, and solvent residues from the extraction process. This approach, therefore, helps to minimize sources of variation observed in previous studies, providing greater reliability and reproducibility to the results obtained.

Among the limitations of this study was the absence of a minimum CADESI-4 inclusion criterion. As several dogs presented with low or null baseline lesion scores, the sensitivity of CADESI-4 to detect treatment-related improvements may have been reduced. However, this approach was intentionally adopted to include dogs representing the full clinical spectrum of canine atopic dermatitis encountered in routine clinical practice. An additional limitation is that pruritus was assessed using the scale proposed by Marsella et al. (18) rather than the validated Pruritus Visual Analog Scale (pVAS), which may limit direct comparison with other studies in the veterinary dermatology literature. Additionally, the relatively small sample size may have limited the statistical power to detect differences in some outcomes. Another limitation was the use of a sequential design, which resulted in complete confounding between treatment and time, rather than a crossover approach. Therefore, the observed changes cannot be clearly distinguished from natural fluctuations in disease severity, regression to the mean, or potential observer expectation bias. Although crossover studies may offer methodological advantages, this design was not adopted due to concerns regarding potential cannabinoid carryover effects and the lack of established washout periods at the time the study was designed. Furthermore, seasonal variation in pruritus was not formally investigated, and therefore it is possible that seasonal fluctuations may have influenced the observed outcomes. Another limitation of this study is the use of a CBD-rich formulation, which prevents evaluation of the potential contribution of THC and minor cannabinoids to the clinical response. In this context, RDC 327/2019 restricts the THC content in Cannabis products marketed in Brazil to a maximum of 0.3% (44). Future studies with formulations with higher THC and other cannabinoids are necessary to further elucidate the therapeutic potential of cannabinoids in cAD.

Based on the results obtained in this study, CBD-rich Cannabis oil represents a promising adjunctive therapeutic approach for cAD. The introduction of cannabinoid therapy was associated with lower pruritus scores compared with baseline values, and no significant worsening of pruritus or skin lesions was observed during the subsequent 30-day period in which the oclacitinib dose was reduced by 50%. These findings suggest that Cannabis oil may represent a promising component of a multimodal therapeutic strategy for the management of cAD. Although the present findings do not support a formal recommendation for early use, they raise the hypothesis that earlier introduction of cannabinoid therapy may provide clinical benefits and warrants further investigation in controlled clinical trials. Randomized controlled studies are needed to establish the specific contribution of Cannabis oil to clinical improvement and to further investigate its potential oclacitinib-sparing effect.

## Data Availability

The original contributions presented in the study are included in the article/Supplementary Material, further inquiries can be directed to the corresponding author.
